# A peptide vaccine targeting angiotensin II attenuates the cardiac dysfunction induced by myocardial infarction

**DOI:** 10.1038/srep43920

**Published:** 2017-03-07

**Authors:** Ryo Watanabe, Jun-ichi Suzuki, Kouji Wakayama, Yasuhiro Maejima, Munehisa Shimamura, Hiroshi Koriyama, Hironori Nakagami, Hidetoshi Kumagai, Yuichi Ikeda, Hiroshi Akazawa, Ryuichi Morishita, Issei Komuro, Mitsuaki Isobe

**Affiliations:** 1 Department of Cardiovascular Medicine, Tokyo Medical and Dental University, Graduate School of Medical and Dental Sciences, Tokyo, Japan; 2 Department of Human Genetics and Disease Diversity, Tokyo Medical and Dental University, Graduate School of Medical and Dental Sciences, Tokyo, Japan; 3 Department of Advanced Clinical Science and Therapeutics, The University of Tokyo, Graduate School of Medicine, Tokyo, Japan; 4 Department of Neurology, Osaka University, Graduate School of Medicine, Osaka, Japan; 5 Department of Health Development and Medicine, Osaka University, Graduate School of Medicine, Osaka, Japan; 6 Department of Cardiovascular Medicine, The University of Tokyo, Graduate School of Medicine, Tokyo, Japan; 7 Department of Clinical Gene Therapy, Osaka University, Graduate School of Medicine, Osaka, Japan.

## Abstract

A peptide vaccine targeting angiotensin II (Ang II) was recently developed as a novel treatment for hypertension to resolve the problem of noncompliance with pharmacotherapy. Ang II plays a crucial role in the pathogenesis of cardiac remodeling after myocardial infarction (MI), which causes heart failure. In the present study, we examined whether the Ang II vaccine is effective in preventing heart failure. The injection of the Ang II vaccine in a rat model of MI attenuated cardiac dysfunction in association with an elevation in the serum anti-Ang II antibody titer. Furthermore, any detrimental effects of the Ang II vaccine were not observed in the rats that underwent sham operations. Treatment with immunized serum from Ang II vaccine-injected rats significantly suppressed post-MI cardiac dysfunction in MI rats and Ang II-induced remodeling-associated signaling in cardiac fibroblasts. Thus, our present study demonstrates that the Ang II vaccine may provide a promising novel therapeutic strategy for preventing heart failure.

The renin-angiotensin system (RAS) plays a pivotal role in the control of blood pressure and cardiovascular physiology. Angiotensin II (Ang), the primary component of RAS, induces hypertension via an Ang II type 1 receptor (AT1R). The chemical drugs that target Ang II, such as angiotensin converting enzyme inhibitor (ACEi) and angiotensin II receptor blocker (ARB), have therefore been widely used as antihypertensive drugs^1^. However, the control of blood pressure is often insufficient due to non-compliance^2^. The increase of the economic burden associated with lifelong medication may be another factor in non-compliance^2^. To resolve these problems and to improve therapeutic effects, a vaccine targeting the RAS was developed as a novel strategy for treating hypertension^3^,^4^. Vaccine treatment is superior due to the duration of its effectiveness in comparison to chemical drugs; it may also be less expensive than conventional medications. Our previous study revealed that treatment with the Ang II vaccine (the conjugate of Ang II and keyhole limpet hemocyanin (KLH)) led to the production of anti-Ang II antibodies and reduced blood pressure in rodent models of hypertension^5^. The harmful effects of Ang II via AT1R induce not only hypertension but also inflammatory, hypertrophic, and fibrotic reactions^6^,^7^. These effects of Ang II are associated with the pathophysiology of cardiovascular disease. Ischemic heart disease, including myocardial infarction (MI), is associated with a high rate of mortality in humans^8^. MI induces morphological changes called remodeling, which leads to heart failure accompanied by infarct area extension and thinning and compensatory hypertrophy of the non-infarcted myocardium^9^,^10^,^11^. Ang II-induced reactions may further damage the myocardium and accelerate post-MI remodeling^12^. In fact, ACEi and ARB have been shown to suppress the progression of post-MI remodeling and improved cardiac function in previous studies^13^,^14^,^15^,^16^. The Ang II vaccine may therefore play a role in preventing heart failure. This study examined whether the Ang II vaccine can effectively prevent cardiac remodeling in a rat MI model.

## Results

### The induction of antibody production by the Ang II vaccine

**Figure 1:**
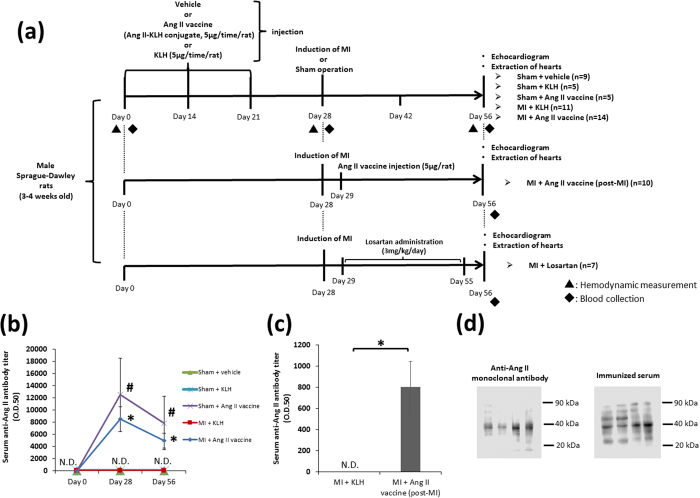
The induction of antibody production by the Ang II vaccine. (**a**) The protocols to investigate the antibody production induced by the Ang II vaccine and the effect of the treatment with the Ang II vaccine. (**b**) The serum anti-Ang II antibody titer. Sham + vehicle, n = 9; Sham + KLH, n = 5; Sham + Ang II vaccine, n = 5; MI + KLH, n = 11; MI + Ang II vaccine, n = 14. *p < 0.05 vs. Sham + KLH; #p < 0.05 vs. MI + KLH. Tukey–Kramer post hoc test. ND, not detected. (**c**) The serum anti-Ang II antibody titer in the MI + Ang II vaccine (post-MI) group. MI + KLH, n = 11; MI + Ang II vaccine (post-MI), n = 10. *p < 0.05 by the Student’s t-test. ND, not detected. (**d**) Comparison of the reactivity of commercial anti-Ang II monoclonal antibody and immunized serum derived from Ang II vaccine-injected rats.

As shown in Fig. 1a, the Ang II vaccine (5 ug/rat) was administered three times to each rat on days 0, 14 and 21. We created the Sham + KLH and MI + KLH groups as control vaccination groups to examine the effect of Ang II vaccine treatment on post-MI remodeling. The Sham + vehicle group was added as normal control group. Additionally, the MI + Ang II vaccine (post-MI) group that received a one-time injection of the Ang II vaccine on the next day after MI induction (day 29) was created to demonstrate the effect of vaccination after MI has occurred. The MI + losartan group was added to compare the treatment effects of the Ang II vaccine and conventional pharmacotherapy (Fig. 1a). To confirm antibody production after Ang II vaccination, we measured the antibody titer against Ang II on days 0, 28, and 56. Although the serum anti-Ang II antibody titer was not detected in any groups on day 0, it was markedly elevated in both the Sham + Ang II vaccine and MI + Ang II vaccine groups on day 28. This elevation of the antibody titer in Ang II vaccine-injected rats was maintained during the experimental period (until day 56) (Fig. 1b). In addition, anti-Ang II antibodies were not detected in the Sham + vehicle, Sham + KLH, and MI + KLH groups during the experimental period (Fig. 1b). The serum anti-Ang II antibody titer in the MI + Ang II vaccine (post-MI) group was significantly elevated on day 56 (Fig. 1c). We performed western blotting using cardiac protein derived from MI rats to confirm the reactivity of the Ang II vaccine-produced antibody (immunized serum), and compared with that of commercial anti-Ang II monoclonal antibody. Commercial anti-Ang II monoclonal antibody detected the band around 38 kDa. Immunized serum which was obtained from Ang II vaccine-injected rats (the Sham + Ang II vaccine group), detected the band at the same place as with commercial anti-Ang II monoclonal antibody, although some extra bands were also detected (Fig. 1d).

### The safety of the Ang II vaccine

**Figure 2:**
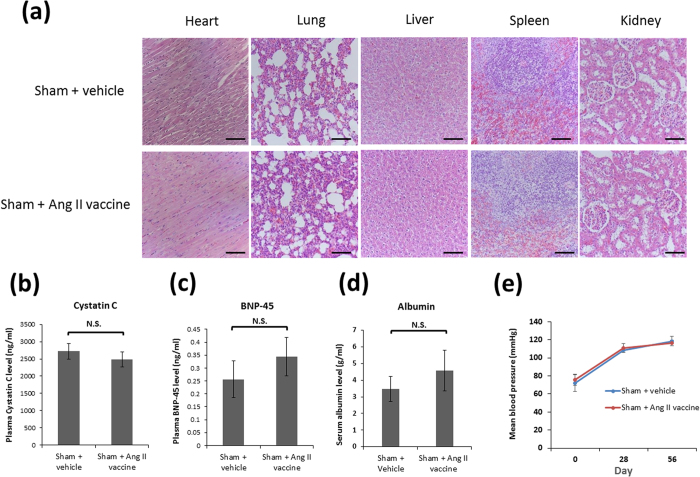
The safety of the Ang II vaccine. (**a**) A representative photomicrograph of hematoxylin-eosin staining in various organs (magnification 200×; scale bars: 100 μm). (**b**) The plasma cystatin C level on day 56. Sham + vehicle, n = 7; Sham + Ang II vaccine, n = 5. NS, not significant. (**c**) The plasma BNP-45 level on day 56. Sham + vehicle, n = 8; Sham + Ang II vaccine, n = 5. (**d**) The serum albumin level on day 56. Sham + vehicle, n = 8; Sham + Ang II vaccine, n = 5. (**e**) The mean blood pressure. Sham + vehicle, n = 5; Sham + Ang II vaccine, n = 5.

We evaluated the effects of the Ang II vaccine not only in MI rats but also in sham-operated rats to examine the safety of the Ang II vaccine (the Sham + Ang II vaccine group), and this was compared with normal rats (the Sham + vehicle group). Visible pathological changes with the injection of the Ang II vaccine in various organs were not observed (Fig. 2a). Next, we measured biomarkers which reflect renal, cardiac, and hepatic function. The injection of the Ang II vaccine did not affect the levels of serum cystatin C (a marker of renal function), plasma brain natriuretic peptide (BNP)-45 (a marker of cardiac function), and serum albumin (a marker of hepatic function) (Fig. 2b–d). Moreover, there was no significant difference in the mean blood pressures between the Sham + vehicle and Sham + Ang II vaccine groups during the experimental period (Fig. 2e).

### The efficacy of the Ang II vaccine on post-MI remodeling

**Figure 3:**
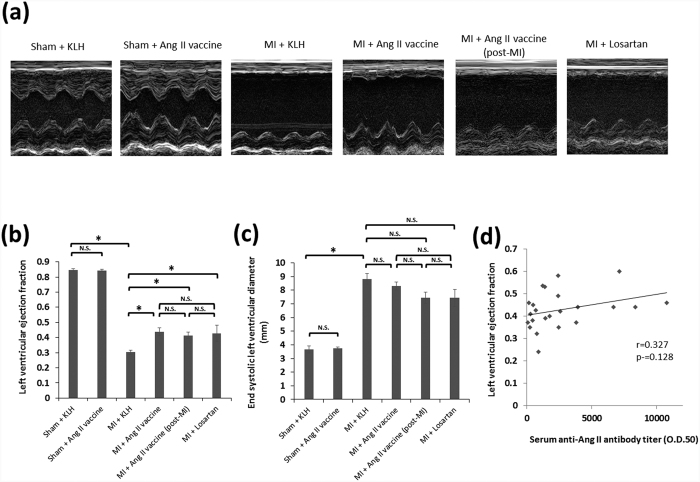
The effects of each of the treatment approaches on cardiac dysfunction after MI. **(a**) Representative M-mode echocardiograms on day 56. A quantitative analysis of (**b**) the left ventricular ejection fraction and (**c**) the end-systolic left ventricular diameter. Sham + KLH, n = 5; Sham + Ang II vaccine, n = 5; MI + KLH, n = 11; MI + Ang II vaccine, n = 13; MI + Ang II vaccine (post-MI), n = 10; MI + losartan, n = 7. *p < 0.05 by the Tukey–Kramer post hoc test. (**d**) The correlation between the left ventricular ejection fraction and the serum anti-Ang II antibody titer in the MI + Ang II vaccine and MI + Ang II vaccine (post-MI) groups.

On day 56, an M-mode echocardiogram showed impaired anterolateral (infarct area) wall motion in the MI + KLH group. The antero-lateral wall motion of the MI + Ang II vaccine, MI + Ang II vaccine (post-MI), and MI + losartan groups remained impaired. However, the regional wall motion of the non-infarct area improved in these groups in comparison to the MI + KLH group (Fig. 3a). In this context, although the left ventricular (LV) ejection fraction significantly declined in the MI + KLH group in comparison to the Sham + KLH group, it significantly improved in all treatment groups of the MI + Ang II vaccine, MI + Ang II vaccine (post-MI), and MI + losartan groups (Fig. 3b). The ischemia-induced dilatation of the LV cavity (as shown by the end-systolic LV diameter) was not significantly suppressed in any of the MI + Ang II vaccine, MI + Ang II vaccine (post-MI), and MI + losartan groups (Fig. 3c). There were no significant differences in the echocardiographic parameters among the MI + Ang II vaccine, MI + Ang II vaccine (post-MI), and MI + losartan groups (Fig. 3b,c). Additionally, the echocardiographic parameters of the sham + KLH and sham + Ang II vaccine groups did not differ to a statistically significant extent (Fig. 3b,c). We further analyzed the correlation between the LV ejection fraction and the serum anti-Ang II antibody titer in the MI + Ang II vaccine and MI + Ang II vaccine (post-MI) groups. However, there was no significant correlation between the LV ejection fraction and the serum anti-Ang II antibody titer (Fig. 3d. r = 0.327, p = 0.128).

**Figure 4:**
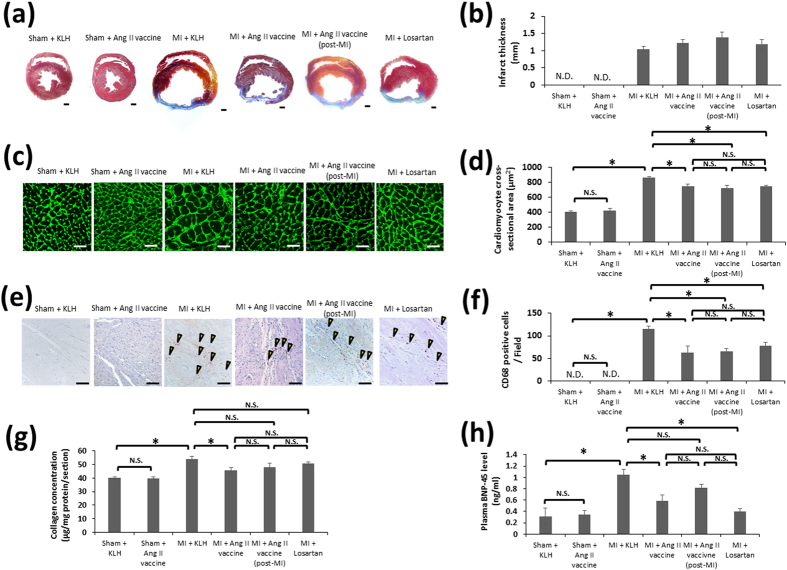
The effects of each of treatment approaches on post-MI remodeling. (**a**) Representative photomicrographs of Mallory-stained cross-sections from each group (magnification, 10×; scale bars: 1 mm) on day 56. (**b**) The quantitative data of the infarct thickness. Sham + KLH, n = 5; Sham + Ang II vaccine, n = 5; MI + KLH, n = 11; MI + Ang II vaccine, n = 14; MI + Ang II vaccine (post-MI), n = 10; MI + losartan, n = 7. (**c**) A representative photomicrograph of the FITC-conjugated WGA staining of the non-infarct area (magnification 400×; scale bars: 50 μm) and (**d**) a quantitative comparison of the cross-sectional size of the cardiomyocytes in each group on day 56. Sham + KLH, n = 4; Sham + Ang II vaccine, n = 4; MI + KLH, n = 6; MI + Ang II vaccine, n = 7; MI + Ang II vaccine (post-MI), n = 6; MI + losartan, n = 7. *p < 0.05 by the Tukey–Kramer post hoc test. (**e**) A representative photomicrograph of CD68 immunostaining (magnification 200×; scale bars: 100 μm) and (**f**) the number of infiltrating macrophages (CD68-positive cells) in the peri-infarct zone on day 56. Sham + KLH, n = 5; Sham + Ang II vaccine, n = 5; MI + KLH, n = 6; MI + Ang II vaccine, n = 6; MI + Ang II vaccine (post-MI), n = 6; MI + losartan n = 7. *p < 0.05 by the Tukey–Kramer post hoc test. (**g**) The cardiac collagen level on day 56. Sham + KLH, n = 4; Sham + Ang II vaccine, n = 5; MI + KLH, n = 8; MI + Ang II vaccine, n = 12; MI + Ang II vaccine (post-MI), n = 7; MI + losartan, n = 6. *p < 0.05 by the Tukey–Kramer post hoc test. (**h**) The plasma BNP-45 level on day 56. Sham + KLH, n = 4; Sham + Ang II vaccine, n = 5; MI + KLH, n = 10; MI + Ang II vaccine, n = 8; MI + Ang II vaccine (post-MI), n = 7; MI + losartan, n = 7. *p < 0.05 by the Tukey–Kramer post hoc test.

Histopathologically, the hearts of the rats in the MI + KLH group showed severe ischemic lesions that were composed of extensive myocardial fibrosis on day 56. Mallory-stained fibrosis (infarcted myocardium) was detected in the anterior wall of the MI + KLH, MI + Ang II vaccine, MI + Ang II vaccine (post-MI), and MI + losartan groups, whereas it was virtually absent in the Sham + KLH and Sham + Ang II vaccine groups (Fig. 4a). The infarct thickness of the MI + KLH, MI + Ang II vaccine, MI + Ang II vaccine (post-MI), and MI + losartan groups did not differ to a statistically significant extent (Fig. 4b). However, the MI-induced hypertrophy of the cardiomyocytes in the non-infarct area was significantly suppressed in all treatment groups: the MI + Ang II vaccine, MI + Ang II vaccine (post-MI), and MI + losartan groups (Fig. 4c,d). Infiltration of CD68-positive macrophages in the peri-infarct zone was observed in the MI + KLH group, whereas it was negligible in the Sham + KLH and Sham + Ang II vaccine groups. The number of infiltrating macrophages was significantly decreased in the all treatment groups of the MI + Ang II vaccine, MI + Ang II vaccine (post-MI), and MI + losartan groups (Fig. 4e,f). There was no significant difference between the Sham + KLH and Sham + Ang II vaccine groups or among the MI + Ang II vaccine, MI + Ang II vaccine (post-MI), and MI + losartan groups in the degree of cardiomyocyte hypertrophy and macrophage infiltration (Fig. 4c–f). The cardiac collagen level, which reflects fibrosis, was elevated in the MI + KLH group in comparison with the Sham + KLH group on day 56. The MI-induced elevation of the cardiac collagen level was significantly suppressed in the MI + Ang II vaccine group. However, it was not significantly suppressed in the MI + Ang II vaccine (post-MI) and MI + losartan groups (Fig. 4g). The plasma BNP-45 level, which is a marker of heart failure, was significantly higher in the MI + KLH group than in the Sham + KLH group on day 56. This elevation of BNP-45 was suppressed in the MI + Ang II vaccine and MI + losartan groups but not in the MI + Ang II vaccine (post-MI) group (Fig. 4h). The cardiac collagen levels and plasma BNP-45 levels among the MI + Ang II vaccine, MI + Ang II vaccine (post-MI), and MI + losartan groups did not differ to a statistically significant extent (Fig. 4g,h). Additionally, the injection of the Ang II vaccine into sham-operated rats did not affect the cardiac collagen and plasma BNP-45 levels (Fig. 4g,h).

### The duration of antibody production induced by the Ang II vaccine

**Figure 5:**
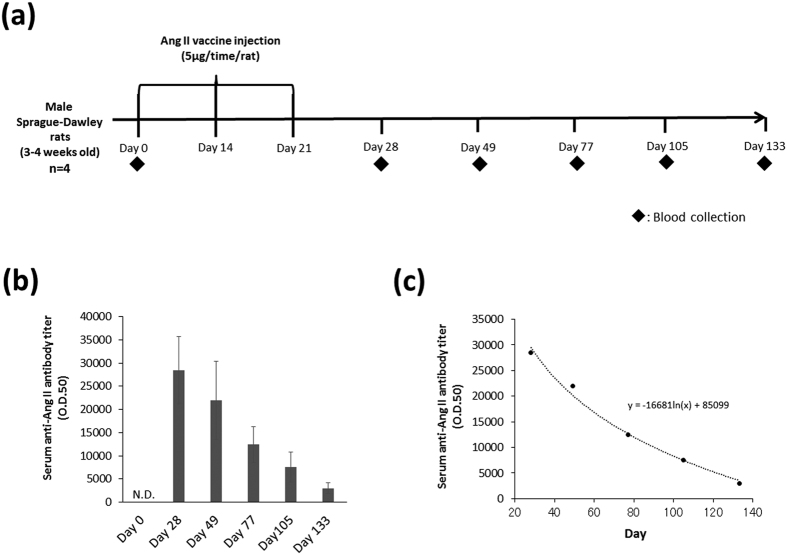
The duration of antibody production induced by the Ang II vaccine. (**a**) The protocol to investigate the duration of antibody production after the administration of the Ang II vaccine. (**b**) The long-term changes in the serum anti-Ang II antibody titer after vaccination. n = 4. (**c**) The exponential decay of the serum anti-Ang II antibody titer.

As shown in Fig. 5a, we measured the serum anti-Ang II antibody titer on days 0, 28, 49, 77, 105, and 133 to examine the half-life and the long-term changes in the antibody titer after vaccination. The anti-Ang II antibody titer was strongly detected on day 28 whereas it was not detected on day 0. After that, the serum anti-Ang II antibody titer decreased gradually (Fig. 5b). The serum half-life of anti-Ang II antibody titer calculated from Fig. 5c was about 42.0 days.

### The evaluation of the potency of the antibody produced by the Ang II vaccine

**Figure 6:**
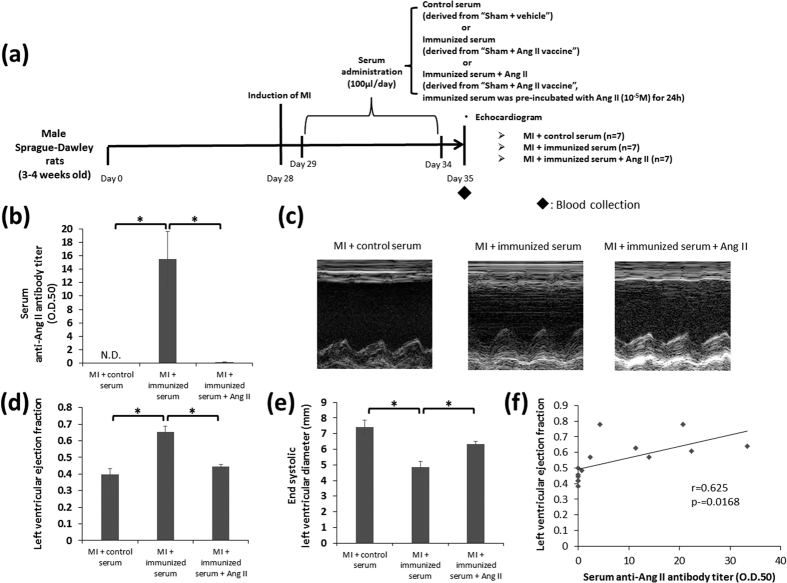
The potency of the antibody produced by the Ang II vaccine on cardiac dysfunction after MI. (**a**) The protocol to evaluate the potency of immunized serum administration on cardiac dysfunction induced by MI. (**b**) The serum anti-Ang II antibody titer. MI + control serum, n = 7; MI + immunized serum, n = 7; MI + immunized serum + Ang II, n = 7. *p < 0.05 by the Tukey–Kramer post hoc test. (**c**) Representative M-mode echocardiograms on day 35. A quantitative analysis of (**d**) the left ventricular ejection fraction and (**e**) the end-systolic left ventricular diameter. MI + control serum, n = 7; MI + immunized serum, n = 7; MI + immunized serum + Ang II, n = 7. *p < 0.05 by the Tukey–Kramer post hoc test. (**f**) The correlation between the left ventricular ejection fraction and the serum anti-Ang II antibody titer in the MI + immunized serum and MI + immunized serum + Ang II groups.

**Figure 7:**
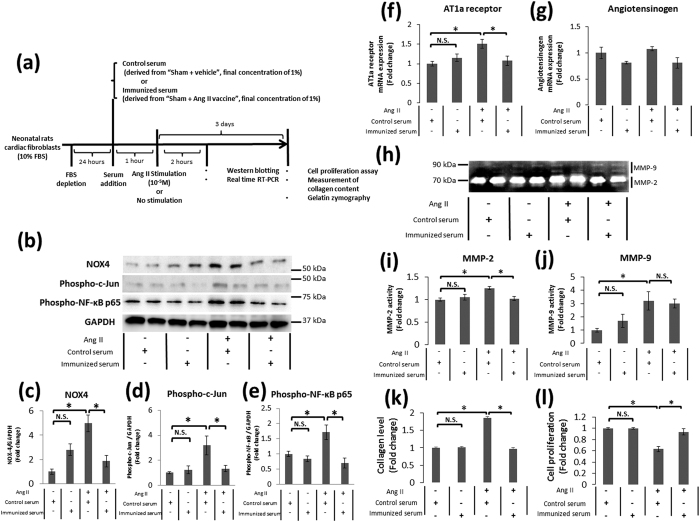
The potency of the antibody produced by the Ang II vaccine on post-MI remodeling-associated responses in cardiac fibroblasts. (**a**) The protocol to evaluate the potency of immunized serum addition on Ang II signaling. (**b**) Representative Western blots of NADPH oxidase 4 (NOX4), phospho-c-Jun, phospho-nuclear factor-κB p65 subunit (NF-κB p65), and glyceraldehyde 3-phosphate dehydrogenase (GAPDH) in neonatal rat cardiac fibroblasts. The relative protein expression of (**c**) NOX4, n = 4 each; (**d**) phospho-c-Jun, n-4 each; and (**e**) phospho-NF-κB p65, n = 5-6 each. *p < 0.05 by the Tukey–Kramer post hoc test. The relative mRNA expression of (**f**) angiotensin type 1a receptor (AT1aR), n = 5-6 each; and (**g**) angiotensinogen, n = 5–6 each. *p < 0.05 by the Tukey–Kramer post hoc test. (**h**) A representative image of gelatin zymography for matrix metalloproteinase (MMP)-2 and MMP-9 in the conditioned media of cultured cardiac fibroblasts. The relative activity of (**i**) MMP-2, n = 6 each; and (**j**) MMP-9, n = 6 each. *p < 0.05 by the Tukey–Kramer post hoc test. (**k**) The relative collagen level in cardiac fibroblasts, n = 12 each; and (**l**) the proliferation ratio of cardiac fibroblasts, n = 12 each. *p < 0.05 by the Tukey–Kramer post hoc test.

To confirm the direct effect of the Ang II vaccine-produced antibody, we daily administered any of control serum derived from vehicle-injected rats, immunized serum derived from Ang II vaccine-injected rats, or immunized serum which was pre-incubated with Ang II (10^−5^M) into unimmunized MI rats (Fig. 6a). The serum anti-Ang II antibody titer was significantly elevated in the MI + immunized serum group whereas it was not detected in the MI + control serum group. Pre-incubation of immunized serum and Ang II decreased the antibody titer to a significantly lower level (Fig. 6b). Treatment with immunized serum significantly improved the LV ejection fraction and suppressed LV dilatation compared with control serum. In contrast, pre-absorption of antibody by Ang II eliminated these cardioprotective effects provided by immunized serum (Fig. 6c–e). A positive correlation was observed between the LV ejection fraction and the serum anti-Ang II antibody titer (r = 0.625, P = 0.0168) in the MI + immunized serum and MI + immunized serum + Ang II groups (Fig. 6f). Ang II is known to activate many factors that accelerate remodeling in cardiac fibroblasts^17^,^18^. Therefore, we next examined whether the Ang II vaccine-produced antibody is able to prevent the Ang II-induced cardiac remodeling-associated reaction in cardiac fibroblasts. Figure 7a shows the *in vitro* experiment protocol. Ang II activates NOX4-AP-1 and NOX4-NF-κB signaling via AT1R^19^. We therefore examined the expression of NOX4, phospho-c-Jun (a component of AP-1), and phospho-NF- κB p65 to determine whether the Ang II vaccine-produced antibody could inhibit Ang II-induced signaling. Western blotting showed that the expression level of NOX4 protein was significantly increased by Ang II stimulation. This increase was inhibited by immunized serum (the final concentration of 1%) but not by control serum (the final concentration of 1%) (Fig. 7b,c). Next, we examined the activation of NF-κB and AP-1 because these transcription factors are a downstream effector of NOX4. The phosphorylation of c-Jun and NF-κB p65 subunit was used as indices of AP-1 and NF-κB activation, respectively. Similarly, the phosphorylation of c-Jun and NF-κB p65 was enhanced by Ang II stimulation. The prior addition of immunized serum to Ang II-stimulated fibroblasts significantly suppressed the phosphorylation of these proteins. In contrast, the control serum did not suppress the phosphorylation induced by Ang II stimulation (Fig. 7b,d,e). Ang II stimulation induces AT1R expression via the activation of transcriptional factors, such as NF-κB or AP-1^20^,^21^, which may cause the positive feedback of RAS activation. Indeed, Ang II stimulation was found to significantly increase AT1R mRNA expression in the present study. This increase was inhibited by treatment with immunized serum but not by treatment with control serum (Fig. 7f). On the other hand, none of the groups showed significant differences in angiotensinogen mRNA expression (Fig. 7g).

Additionally, the activation of AP-1 and/or NF-κB is involved in the progression of cardiac remodeling by the induction of MMP activation and collagen synthesis in cardiac fibroblasts^19^. We therefore analyzed the collagen level in fibroblasts and MMP activity in culture supernatants after Ang II stimulation. Gelatin zymography showed that Ang II stimulation induced MMP-2 and MMP-9 activation. The increased MMP-2 activity was only attenuated by immunized serum; the MMP-9 activity was not altered (Fig. 7h–j). Moreover, the addition of immunized serum suppressed the increase in the collagen level induced by Ang II in comparison to control serum (Fig. 7k). The proliferation of cardiac fibroblasts was decreased three days after Ang II stimulation. Treatment with immunized serum eliminated the Ang II-induced reduction of cellular proliferation; this effect was not observed with control serum (Fig. 7l). The addition of immunized serum in the absence of Ang II stimulation did not induce any reactions in the cardiac fibroblasts (Fig. 7b–l).

## Discussion

In the present study, we demonstrated, for the first time, that a peptide vaccine targeting Ang II (Ang II-KLH conjugate) attenuated the cardiac remodeling that leads to heart failure, improving cardiac function and suppressing adverse pathological changes in a rat model of MI. The antigen must be recognized by both B-cells and T-cells to induce antibody production. However, Ang II does not have a T-cell epitope. Thus, antibodies against Ang II are not usually produced. For this reason, Ang II peptide was conjugated with the KLH of a carrier protein with both B-cell and T-cell epitopes^22^,^23^. A previous study revealed that this conjugate, defined as the Ang II vaccine, induced the production of anti-Ang II antibodies, and led to a therapeutic effect in animal models of hypertension^5^.

As expected, the serum anti-Ang II antibody titer was markedly elevated in Ang II vaccine-injected rats whereas it was negligible in unimmunized rats. We confirmed the reactivity of immunized serum by the Ang II vaccine in cardiac protein from MI rats based on the evidence that Ang II is abundant in ischemic heart tissue^12^,^24^. The band detected by immunized serum was compared with that of commercial anti-Ang II monoclonal antibody. Western blotting using commercial anti-Ang II monoclonal antibody detected the band around 38 kDa. This result is consistent with that of previous studies using the same antibody^25^,^26^. In contrast, immunized serum detected some extra bands in addition to the band around 38 kDa. This result may be attributed to the difference of specificity between monoclonal antibody and antiserum. Thus, immunized serum may have the reactivity to not only Ang II but also other proteins owing to that specificity. In this study, we also studied the safety of the Ang II vaccine using sham-operated rats because there were the concerns that the Ang II vaccine-produced antibody may affect the proteins other than Ang II and Ang II induces organ damage and hypertension^6^.

The injection of the Ang II vaccine did not affect the blood pressure in rats. Additionally, the level of biomarkers which reflect cardiac, renal, and hepatic function was not changed in Ang II vaccine-injected rats compared with that of normal rats. After the injection of the Ang II vaccine, harmful effects on cardiac function and histopathological changes in various organs were not observed. These results suggest the Ang II vaccine did not have obvious toxic effects.

We induced MI in rats after the injection of the Ang II vaccine to examine the effectiveness of the Ang II vaccine as a preventive therapy. Our results showed that the prior injection of the Ang II vaccine was found to suppress the decline in cardiac function, inflammation, the increase in the cardiac collagen level, along with compensatory hypertrophy, which are typical features of post-MI remodeling. Similarly, the elevation of the plasma BNP-45 level after MI was significantly attenuated. These results may suggest the effectiveness of the Ang II vaccine in the prevention of heart failure.

We also examined the effect of the injection of the Ang II vaccine after infarct occurrence to investigate the additional utility of the Ang II vaccine in clinical practice. Although there was no statistical difference between the MI + KLH and MI + Ang II vaccine (post-MI) groups regarding cardiac collagen concentration and plasma BNP level, post-MI injection of the Ang II vaccine improved the LV ejection fraction and suppressed cardiomyocyte hypertrophy and macrophage infiltration. These results suggest that the Ang II vaccine treatment partly provided cardioprotective effects even when the injection was performed after MI. Prior-injection of the Ang II vaccine may be more effective, and may be beneficial as secondary prevention in the patient who has a high risk of heart failure such as hypertension. Consistent with the results of many previous studies^15^,^27^, losartan treatment attenuated post-MI remodeling and improved cardiac function. The treatment effects among the MI + Ang II vaccine, MI + Ang II vaccine (post-MI), and MI + losartan groups did not differ to a statistically significant extent in our rat model of MI. This result suggests that the injection of the Ang II vaccine may be as effective as taking losartan daily after MI. LV cavity dilatation and thinning of the infarcted myocardial wall induced by ischemia were not significantly attenuated by both Ang II vaccine and losartan treatment. In some previous studies, anti-Ang II treatment such as ARB or ACEi did not completely improve these remodeling-associated harmful changes^28^,^29^. Generically, it is considered that not only Ang II but also many other factors such as proinflammatory cytokines, hormones, and growth factors are involved in the progression of cardiac remodeling after MI^18^,^30^. We hypothesize that, although Ang II vaccine treatment does not suppress the progression of post-MI remodeling completely, it is effective for improvement of the outcome after MI.

We next examined the long-term changes of the serum anti-Ang II antibody titer after the injection of the Ang II vaccine. The serum half-life of anti-Ang II antibody titer was about 42.0 days. Moreover, anti-Ang II antibodies were detected even on day 133. The major weak point of pharmacotherapy as traditional treatment is the shortness of the duration of action. Hence, the patient must take medicine every day. Additionally, the long-term treatment of the disease is costly^2^,^23^. These problems may lead to non-compliance^3^. Thus, Ang II vaccine treatment may have an advantage over conventional pharmacotherapy with ACEi or ARB regarding its duration of action.

We next daily administered immunized serum derived from the Sham + Ang II vaccine group into unimmunized MI rats to confirm the treatment effects of the Ang II vaccine on cardiac dysfunction associated with MI. Treatment with immunized serum significantly improved the LV ejection fraction in MI rats. On the other hand, pre-incubation with Ang II eliminated the cardioprotective effect of immunized serum. Thus, it was suggested that MI-induced cardiac function decline was prevented by the Ang II vaccine-produced antibody. Furthermore, we examined the potency of the Ang II vaccine-produced antibody against the Ang II-induced cellular response using rat neonatal cardiac fibroblasts. Ang II induces the activation of NF-κB and AP-1 via AT1R and NOX4^19^. Activated NF-κB and AP-1 cause responses that promote post-MI remodeling, including positive feedback of the activation of the RAS, MMP activation, and the promotion of collagen synthesis via the induction of gene expression^17^,^19^,^31^,^32^,^33^,^34^,^35^. The Ang II-induced expression of AT1R mRNA, phospho-c-Jun, phospho-NF-κB, and NOX4 were inhibited by treatment with immunized serum. Similarly, immunized serum attenuated the MMP-2 activation and collagen production induced by Ang II. Ang II stimulation reduced the proliferation of cardiac fibroblasts in this study. We hypothesize that this result is attributed to the apoptosis-inducing effect of Ang II^36^,^37^. The Ang II-induced reduction of cellular proliferation was counteracted by treatment with immunized serum. Consequently, it was suggested that the Ang II-induced cardiac remodeling-associated reaction was inhibited by the Ang II vaccine-produced antibody. The results of the *in vivo* and *in vitro* studies that examined the direct effect of the Ang II vaccine-produced antibody support the cardioprotective effects of the Ang II vaccine in a rat model of MI.

A previous study reported that a negative correlation was observed between the serum anti-Ang II antibody titer and blood pressure in Ang II-induced hypertension^5^. On the basis of this result, we evaluated whether there is antibody titer-dependent variation in the cardioprotective effect of the Ang II vaccine. However, a significant correlation was not observed between the LV ejection fraction and the serum anti-Ang II antibody titer in Ang II vaccine-injected MI rats. On the other hand, cardiac function decline was attenuated in immunized serum-injected MI rats despite a relatively low anti-Ang II antibody titer. Moreover, there was a significant positive correlation between the LV ejection fraction and the serum anti-Ang II antibody titer in the treatment with immunized serum. Thus, we speculate that the neutralization effect of the Ang II vaccine produced-antibody to Ang II reached a plateau, and a large amount of antibody is not always necessary for the neutralization of Ang II increased by MI.

Our present study revealed that Ang II vaccine treatment improved the pathophysiology after MI. The Ang II vaccine may therefore reduce the risk of heart failure in addition to improving hypertension. We conclude that Ang II vaccine treatment may be beneficial as a new approach for preventing heart failure that solves the problem of non-compliance to conventional pharmacotherapy.

## Materials and Methods

### The preparation of the Ang II vaccine peptide

The N-terminus of the human Ang II peptide and KLH of the carrier protein was conjugated using glutaraldehyde as a condensing agent as described previously^5^. This conjugate was synthesized in the Peptide Institute Inc. (Osaka, Japan), and was used as the Ang II vaccine in the subsequent experiments.

### The *in vivo* experiment protocol

Figures 1a, 5a, and 6a show the protocol of the *in vivo* experiments.

Male Sprague-Dawley rats (3-4 weeks old) were purchased from CLEA Japan, Inc. (Tokyo, Japan). The rats were anesthetized by the combination of 0.375 mg/kg medetomidine, 2.0 mg/kg midazolam, and 2.5 mg/kg butorphanol with the intraperitoneal (i.p.) administration immediately before the procedure. The Ang II vaccine was dissolved in saline, and emulsified with an equal volume of Freund’s adjuvant (Wako Pure Chemical Industries). One hundred microliters of the emulsion was subcutaneously injected into two sites on the rats (5 μg vaccine peptide/200 μl emulsion).

As shown in Fig. 1a, the vaccination was performed a total of three times on days 0, 14, and 21. Complete Freund’s adjuvant was used in the first immunization (day 0), and incomplete Freund’s adjuvant was used in second and third immunizations to boost the immune response (days 14 and 21). We also used unimmunized rats in a parallel manner. Unimmunized rats were injected with vehicle (saline) or the emulsion of KLH and adjuvant. In rats of the MI + KLH, MI + Ang II vaccine, MI + Ang II vaccine (post-MI), and MI + losartan groups, myocardial infarction (MI) was induced by ligating the left anterior descending (LAD) coronary artery on day 28. In rats of the Sham + vehicle, Sham + KLH, and Sham + Ang II vaccine groups, sham operation was performed without LAD ligation. In the MI + Ang II vaccine (post-MI) group, the injection of the emulsion of the Ang II vaccine and complete Freund’s adjuvant was performed once on the next day after MI induction (day 29). We also created an MI + losartan group to compare the treatment effects. Losartan was purchased from Wako Pure Chemical Industries, Ltd. (Osaka, Japan). Losartan was dissolved in 0.5% carboxymethylcellulose (CMC) solution immediately before use, and it was orally daily administered to unimmunized MI rats from day 29 to day 55. The dose of losartan (3 mg/kg/day) was determined according to previous studies^15^,^27^. Blood samples were collected from the femoral vein on days 0, 28, and 56. After the centrifugation of the blood samples, the supernatant (serum or plasma) was stored at −80 °C until use. Rats were sacrificed after echocardiography on day 56.

In the evaluation of the half-life and long-term change in the serum anti-Ang II antibody titer (Fig. 5a), rats were immunized with the Ang II vaccine in the same manner as shown in Fig. 1a. Blood samples (serum) were collected on days 0, 28, 49, 77, 105, and 133 from normal rats (without MI) that were immunized with the Ang II vaccine. The half-life of the serum anti-Ang II antibody titer was calculated from the curve fitting of exponential decay as shown in Fig. 5c.

In the evaluation of the potency of the Ang II vaccine-produced antibody (Fig. 6a), MI was induced on day 28. Subsequently, we daily administered any of control serum (serum derived from the Sham + vehicle group on day 28), immunized serum (serum derived from the Sham + Ang II vaccine group on day 28), and immunized serum which was pre-incubated with Ang II (10^−5^ M) for 24 h to unimmunized MI rats by injection into the femoral vein from day 29 to day 34. The concentration of Ang II was determined from the data of the binding ability of the Ang II vaccine-produced antibody to Ang II as described in our previous study^5^. These rats were then subjected to echocardiography on day 35. All of the animal experiments were approved by the Institutional Animal Care and Use Committee of Tokyo Medical and Dental University. These experiments were conducted according to the National Research Council’s guidelines.

### Hemodynamic Measurements

A tail-cuff system (BP-98A, Softron Co., Tokyo, Japan) was used to measure the blood pressure of the rats on days 0, 28, and 56. The rats were adapted to the apparatus for at least five days before the study was initiated.

### Echocardiography

Transthoracic echocardiography was performed on the rats after they were anesthetized on day 56 in Fig. 1a or on day 35 in Fig. 6a. An echocardiography machine with a 7.5-MHz transducer (“Nemio”, Toshiba Co., Tokyo, Japan) was used for M-mode left ventricular echocardiographic recording. A 2D targeted M-mode echocardiogram was obtained along the short-axis view of the left ventricular papillary muscles. The LV ejection fraction and end-systolic LV diameter were calculated from the M-mode recordings.

### Histopathological examination

Hearts were harvested after the echocardiogram examination on day 56 (Fig. 1a). The hearts were divided into apex, midventricular (under the ligation point) and basal level slices. A midventricular level slice was fixed with formalin. Formalin-fixed paraffin sections were used in the histopathological examinations, the immunohistochemical analyses, and in the measurement of collagen concentration. Additionally, the samples of the lung, liver, spleen, and kidney harvested from the Sham + vehicle and Sham + Ang II vaccine groups were stained with hematoxylin-eosin after formalin fixation to confirm presence or absence of adverse pathological changes by the Ang II vaccine. The degree of cardiac hypertrophy in the non-infarct area after MI was estimated by fluorescein isothiocyanate-conjugated wheat germ agglutinin (WGA). After WGA staining, we randomly measured 100 myocytes per sample section, and calculated the average cross-sectional area of the myocytes^38^,^39^. Mallory staining was used to estimate the area of infarcted myocardium (consisting of myocardial fibrosis). Infarct thickness was measured in five places at equal intervals along the infarct area; the value was then averaged. These histopathological analyses were performed using a computer-assisted analyzer (“Scion Image Beta 4.0.2”, Scion Corporation, Frederick, MD, USA).

### Immunohistochemistry

Immunohistochemistry was performed using a midventricular level slice on day 56 (Fig. 1a) to examine the degree of macrophage infiltration after MI. Paraffin sections were incubated overnight at 4 °C with a primary antibody against CD68 (ED1, AbD serotec, Oxford, UK). Subsequently, sections were washed with phosphate-buffered saline (PBS), and were incubated with a secondary antibody (“Histofine Simple Stain Rat”, Nichirei Co., Tokyo, Japan) for 30 minutes at room temperature. Finally, the sections were reacted with aminoethyl carbazole complex (AEC) solution (Nichirei Co.) for 5 to 30 minutes. We counted the number of CD68-positive cells in four randomly selected microscopic fields (original magnification, ×200) from the peri-infarct zone (the border zone between the infarct and non-infarct areas) in each sample section; the value was then averaged.

### The measurement of the antibody titer

Bovine serum albumin (BSA) was conjugated to the peptide of Ang II at the Peptide Institute Inc. (Osaka, Japan). The serum anti-Ang II antibody titer was measured using this conjugate as described previously^5^. Briefly, Ang II-BSA conjugate was dissolved in a carbonate buffer (10 μg/ml), and coated on a 96-well plate. The coated plates were incubated overnight at 4 °C. After blocking using 5% skim milk solution, the samples were diluted from 10-fold to 1,000,000-fold with a 5% skim milk solution, and were incubated in the plate overnight at 4 °C. The plates were then washed and rat IgG horseradish peroxidase (HRP)-conjugated antibody was added. The plates were incubated for 3 hours at room temperature and then washed and the antigen-antibody reaction was detected using 3,3′,5,5′-tetramethylbenzidine (TMB) solution and 0.5 N sulfuric acid. The absorbance of the solution at 450 nm was measured. The antibody titer was evaluated from the absorbance using the sample dilution range as the half-maximum binding titer (OD50).

### The measurement of biomarkers which reflect renal, cardiac, and hepatic function

Plasma or serum samples (from day 56) were used to measure the level of cystatin C, BNP-45, and albumin as biomarkers of renal, cardiac, and hepatic function. The concentrations of cystatin C (“Mouse/Rat Cystatin C Quantikine ELISA Kit”, R&D Systems, Minneapolis, MN, USA), BNP-45 (“Rat BNP-45 ELISA Kit”, Assaypro, St. Charles, MO, USA), and albumin (“Rat Albumin ELISA Quantitation Set”, R&D Systems) were determined using an enzyme-linked immunosorbent assay according to the manufacturer’s instructions.

### Cell preparation

Sample hearts were obtained from 2- to 4-day-old neonatal Wistar rats, and were resolved by collagenase (Roche Diagnosis). Cardiac fibroblasts were then isolated as described previously^40^,^41^, and were cultured in Dulbecco’s Modified Eagle Medium containing 10% fetal bovine serum (FBS).

### The *in vitro* experiment protocol

Figure 7a shows the *in vitro* experiment protocol in the evaluation of the potency of the antibody produced by the Ang II vaccine. FBS was removed from a culture medium of fibroblasts. Twenty-four hours after FBS depletion, the cells were pre-incubated with immunized serum (serum derived from the Sham + Ang II vaccine group on day 28, the final concentration of 1%) or control serum (serum derived from the Sham + vehicle group on day 28, the final concentration of 1%) for 1 hour at 37 °C. The cells were then stimulated with or without Ang II (10^−5^ M) (Sigma-Aldrich), and were incubated at 37 °C under 5% CO_2_. *In vitro* dosage of Ang II was determined based on previous studies^42^,^43^,^44^. The sample for each assay was obtained at 2 hours or 3 days after Ang II stimulation.

### The measurement of the collagen concentration

The concentration of collagen in the paraffin tissue sections and cultured fibroblasts was measured using a “Sirius Red/Fast Green Collagen Staining Kit” (Chondrex Inc, Redmond, US) according to the manufacturer’s instructions. Briefly, collagen proteins were stained with Sirius Red, and non-collagenous proteins were stained with Fast Green. Subsequently, the dye was eluted from the samples with dye extraction solution. The absorbance of the solutions was measured at 540 or 605 nm, respectively. The collagen concentration was calculated from the absorbance. The concentration of collagen per tissue section was calculated as the ratio of collagen to total protein^45^,^46^. The data from cultured cardiac fibroblasts were normalized as the fold change to the control (control serum without Ang II stimulation).

### Cell proliferation assay

At 72 hours after Ang II stimulation, the cells were centrifuged at 200 × *g* for 5 min. Culture supernatants were stored after the measurement of protein concentration until use in gelatin zymography at −80 °C. The degree of cell proliferation was determined based on precipitation using a “Cell Counting Kit-8” (Dojindo, Tokyo, Japan) according to the manufacturer’s instructions. Cell proliferation was expressed as the optical density, and normalized as the fold-change to the control.

### Protein extraction

To extract cellular protein, cardiac fibroblasts (2 hours after Ang II stimulation) were lysed with “M-PER Mammalian Protein Extraction Reagent” (Thermo Scientific) containing an EDTA-free protease inhibitor cocktail tablet (Roche Diagnostic, Basel, Switzerland) and a phosphatase inhibitor tablet (Roche Diagnostic, Basel, Switzerland). For extraction of cardiac protein, frozen peri-infarct zone tissues from apex-level heart slices harvested from the MI + KLH group on day 56 were homogenized in lysis buffer (50 mM Tris–HCl (pH 7.5), 150 mM NaCl, 1% Triton X-100, 1% sodium deoxycholate, 1% sodium dodecyl sulfate (SDS)) containing a protease inhibitor cocktail tablet. The lysates were centrifuged at 14000 × *g* for 15 minutes. The protein concentrations of all samples including culture supernatant were measured using the “Bio-Rad Protein Assay Kits” (Bio-Rad, Milan, Italy) to equalize the protein concentrations. These protein samples were stored at −80 °C, and were used as samples for Western blotting and gelatin zymography.

### Gelatin zymography

Gelatin zymography was performed using protein samples from culture supernatants as described previously^47^. Briefly, Ten microliters of protein sample was mixed with an equal volume of 2× loading buffer (0.5 M Tris–HCl [pH 6.8], 20% glycerol, 4% SDS, and 0.05% bromophenol blue) and incubated for 10 minutes at room temperature. The samples were loaded onto a 6% gelatin gel, and were separated by sodium dodecyl sulfate- polyacrylamide gel electrophoresis (SDS-PAGE) at 150 V. The gel was incubated in a “Novex Zymogram Renaturing Buffer” (Invitrogen Corp, Carlsbad, CA) for 30 minutes, and replaced with a “Novex Zymogram Developing Buffer” (Invitrogen) followed by incubation at 37 °C for 24 hours. The gel was stained with 0.2% Coomassie blue for 2 hours and destained. MMP-9 and MMP-2 were expressed as clear bands against the background; this was attributable to enzyme activity. MMP activity was quantified by the densitometry of these bands.

### Western blotting

Ten microliters of protein sample was mixed with an equal volume of 2× SDS sample buffer [0.5 M Tris–HCl (pH 6.8), 20% glycerol, 4% SDS, 0.05% bromophenol blue] and incubated for 5 min at 95 °C. The samples were separated by SDS-PAGE with a 10% acrylamide gel at 150 V, then transferred to a nitrocellulose membrane. The membranes were incubated overnight at 4 °C with any of the following primary antibodies: glyceraldehyde 3-phosphate dehydrogenase (1:5000, GAPDH, Cell Signaling Technology, Danvers, MA, USA), phospho-NF-κB p65 (1:1000, Cell Signaling Technology), phospho-c-Jun (1:1000, Cell Signaling Technology), NADPH oxidase 4 (1:2000, Abcam, Cambridge,UK), angiotensin II (1:1000, monoclonal antibody, Novus, Littleton, CO, USA), and immunized serum derived from Ang II vaccine-injected rats (the Sham + Ang II vaccine group on day 28) (1:1000). The membranes were then incubated with a secondary antibody (1:20000, GE Healthcare, Buckinghamshire, UK) for 1 hour, and developed using a “Super Signal West Dura Extended Duration Substrate” (Thermo Scientific). Enhanced chemiluminescence was detected with a “LAS-1000” (Fujifilm, Tokyo, Japan). The value was calculated using “Image J” software program (National Institutes of Health [NIH]).

### Real-time RT-PCR

“TRIsure” (BIOLINE, London, UK) was used to isolate total RNA from cardiac fibroblasts at 2 hours after Ang II stimulation. cDNA was obtained using a “High Capacity cDNA Reverse Transcription Kit” (Applied Biosystems, Inc., California, USA) from this RNA. A “Step One Real-Time PCR System” (Applied Biosystems) was used to measure the mRNA expression of AT1aR (Assay ID: Rn02758772_s1), angiotensinogen (Assay ID: Rn00593114_m1), and 18 s rRNA (Assay ID: Hs99999901_s1, as a control). cDNA was run in duplicate, and quantitative data were calculated using the comparative CT (ΔΔCT) method. The mRNA expression was normalized as the fold change to the control (control serum without Ang II stimulation)^48^,^49^.

### Statistical Analysis

All data are expressed as the mean ± SEM. Statistical analyses were performed using the “BellCurve for Excel” software program (Social Survey Research Information Co., Ltd., Tokyo, Japan). The data among multiple groups were analyzed using a one-way analysis of variance (ANOVA) followed by the Tukey–Kramer method. The Student’s t-test was used to compare data between two groups in Figs 1c and 2b–e. P values of < 0.05 were considered to indicate statistical significance.

## Additional Information

**How to cite this article:** Watanabe, R. *et al*. A peptide vaccine targeting angiotensin II attenuates the cardiac dysfunction induced by myocardial infarction. *Sci. Rep.* **7**, 43920; doi: 10.1038/srep43920 (2017).

**Publisher's note:** Springer Nature remains neutral with regard to jurisdictional claims in published maps and institutional affiliations.
